# Mucosal Immunity in the Female Genital Tract, HIV/AIDS

**DOI:** 10.1155/2014/350195

**Published:** 2014-09-15

**Authors:** Juliana Reis Machado, Marcos Vinícius da Silva, Camila Lourencini Cavellani, Marlene Antônia dos Reis, Maria Luiza Gonçalves dos Reis Monteiro, Vicente de Paula Antunes Teixeira, Rosana Rosa Miranda Corrêa

**Affiliations:** ^1^Discipline of General Pathology, Nephropathology Service, Federal University of Triangulo Mineiro, Rua Frei Paulino n° 30, Bairro Abadia, 38025-180 Uberaba, MG, Brazil; ^2^Discipline of Immunology, Federal University of Triangulo Mineiro, Rua Frei Paulino n° 30, Bairro Abadia, 38025-180 Uberaba, MG, Brazil

## Abstract

Mucosal immunity consists of innate and adaptive immune responses which can be influenced by systemic immunity. Despite having been the subject of intensive studies, it is not fully elucidated what exactly occurs after HIV contact with the female genital tract mucosa. The sexual route is the main route of HIV transmission, with an increased risk of infection in women compared to men. Several characteristics of the female genital tract make it suitable for inoculation, establishment of infection, and systemic spread of the virus, which causes local changes that may favor the development of infections by other pathogens, often called sexually transmitted diseases (STDs). The relationship of these STDs with HIV infection has been widely studied. Here we review the characteristics of mucosal immunity of the female genital tract, its alterations due to HIV/AIDS, and the characteristics of coinfections between HIV/AIDS and the most prevalent STDs.

## 1. Introduction

The relationship between STDs and HIV infection has been widely studied. At the end of 2012, approximately 35.13 million people were infected with HIV worldwide. The average prevalence of coinfection between HIV and genital inflammatory diseases is of 16.3% [1].

## 2. Immune Cells of the Female Genital Mucosa

The immune system of the female genital tract is part of the integrated mucosal immune system, but with some particular characteristics that differentiate the immunity of these regions from the systemic immunity [2–4]. Mucosal immunity is related to its own function, such as maintenance of embryonic development during pregnancy and female reproductive organ functioning during copulation; when in contact with the external environment, the lower portion of the female genital tract is susceptible to various microorganisms. This portion of the female genital tract comprises the vagina and the ectocervix, and it has a commensal microbiota that consists predominantly of* Lactobacillus *[5]. The upper portion of the female reproductive tract consists of the fallopian tubes, uterus body, and endocervix, which has columnar epithelial cells and is distincted from ectocervix which is part of the lower tract characterized by squamous epithelial cells [4, 6]. The human female reproductive tract (FRT) is not an immunologically sterile but rather an immunologically active site [7–12].

Many studies have shown the upper FRT to be immunologically viable and responsive. Recent reports have demonstrated that TLRs 7–9 are constitutively expressed in fallopian tubes, uterine endometrium, cervix, and ectocervix, while expression of TLR10 is restricted to the fallopian tubes. NOD1 and NOD2 as well as the signal transducer RICK are detectable in all FRT tissues. Moreover, these receptors are functional, as treatment of FRT tissue cells with ligands for TLR and NOD induces production of proinflammatory CXCL8 [13], and those receptors actively participate in immune response to pathogens, as* Neisseria gonorrhea* and HIV-1 [14].

Mucosal immunity consists of innate and adaptive immune responses that can be influenced by systemic immunity [15] and by hormonal changes during the menstrual cycle. Hormones regulate the immune system throughout the female reproductive tract in a way that favors conditions for sperm migration, fertilization, implantation, and pregnancy [16, 17].

Innate immunity includes barriers such as the epithelium, mucus, pH, complement system, and cells of the immune system. The squamous epithelium of vagina and ectocervix recovers the majority of the exposed surface area of the FRT mucosa. It comprises a significant physical barrier to small molecule forms of ingress, such as HIV, due to the thick multilayered structure [18]. In this epithelium, as well as in the more fragile single cell layer epithelium of endocervix, the cells are held together by proteins that form desmosomes, tight junctions, and adherens junctions, which decrease its permeability [19]. For some, this is an impenetrable barrier for agents such as HIV, but Langerhans cells within the squamous layer have been shown to transmit the virus for target cells [20]. In addition, CD4^+^ cells infiltrating the epithelium can act as potential target cells to initiate transmission [21].

The mucus is comprised of mucins which form a very thick gel that functions as a physical barrier to pathogens [22, 23]. Its aqueous part, rich in immunoglobulins and in antimicrobial peptides, is another form of protection [24]. This barrier is important to protect the upper tract from ascending infections.

A major component of the mucus that affects pathogen transmission is the pH. The pH is maintained by the local presence of commensal bacteria, which keep the pH acidic through the production of lactic acid and hydrogen peroxide, H_2_O_2_, which has antimicrobicidal activity [5, 25]. Together, the epithelial cells, mucus, lactic acid produced by commensal bacteria, and proteins of the complement system form a dynamic physiological structure that interacts with microorganisms to prevent infections [15].

Macrophages and dendritic cells (DCs) are important cells which phagocyte and destroy pathogens by acid and enzyme digestion. The macrophages in the female reproductive tract are more concentrated in the endometrium and in the myometrial connective tissue [10]. In the endometrium, they are regulated by estradiol and progesterone [26]. In the vagina, the number of macrophages remains stable throughout the menstrual cycle [10].

DCs are located in the endometrial subepithelial stroma, whereas vaginal DCs are found in the epithelial layer [27]. It was recently demonstrated that uterine epithelial cells secrete soluble mediators to the stroma and that these mediators can induce a tolerogenic phenotype in local dendritic cell populations. This phenotype is characterized by a decrease in the expression of CD83 and CD86 costimulatory molecules and by a decrease in TLR3 and TLR4 stimulation and sensitivity stimulation [28].

NK cells consist of approximately 70% of leukocytes in the endometrial mucosa and these cells have phenotypic characteristics which are different from NK cells in the blood, as they express markers such as CD9, CD69, and CD94 [29]. Uterine NK cells promote a local inflammatory response through the production of proinflammatory cytokines and chemokines, such as GM-CSF, IL-10, IL-8, and IFN-*γ*, thus inducing macrophage activation and generation of cytotoxic T-cells. However, it is believed that the most important role of these cells concerns the defense against viruses, especially herpes [30].

Neutrophils are present throughout the FRT; they are found in larger quantities in the fallopian tubes and progressively decrease from the upper reproductive tract to the vagina [10]. Neutrophil count is relatively constant throughout the menstrual cycle. However, there is a significant increase in neutrophils in the endometrium during menstruation, which is preceded by an increase in IL-8. The presence of neutrophils may serve two purposes in menstruation; firstly, it assists in endometrial tissue destruction via elastase release, which subsequently activates extracellular matrix metalloproteinases; secondly, it increases innate immune defense, since the epithelial barrier is interrupted. Neutrophils express TLRs 1–9 and respond to pathogens through phagocytosis, production of oxidative compounds, and release of antimicrobial peptides. It is known that neutrophils produce protease inhibitors (Trappin-2/Elafin), *α*-defensins known as human neutrophil peptides (HNPs), phospholipases, and cytokines [31].

Innate immune response in the female reproductive system is regulated by cytokines and chemokines. Type I interferons (IFNs) are an important cytokine family involved in female reproductive tract immunity, especially against viruses, and are composed of the following subtypes in humans: IFN*α*, IFN*β*, IFN*ε*, IFN*ω*, and IFN*κ*. IFNs are rapidly induced in the presence of viruses and bacteria [7, 32]. Although the role of type I IFNs in innate immune response is well defined, the role of hormones in the modulation of these cytokines is still not well elucidated [15]. In most cases, secretion of chemokines is important to attract immune cells to the epithelial surface [8]. Among the cytokines involved, IL-8 and TGF-beta play an important role as they seem to influence the development and function of local immune cells [7, 8, 28, 33]. Furthermore, the levels of these cytokines are influenced by hormones during the menstrual cycle, particularly in cervicovaginal region [34]. In acute HIV infections, there is an increase in genital tract cytokine levels, both those with proinflammatory actions like IL-6 and IL-12 and those with anti-inflammatory ones, as IL-10 [35]. The elevated levels of local proinflammatory cytokines are related to HIV shedding [36]. IL-1*β*, a proinflammatory cytokine produced by macrophages, and IL-8, a chemokine produced by epithelial cells, macrophages, and other cells, were significantly associated with HIV-RNA in cervicovaginal lavage independent of plasma viral load [37]. Thus, the systemic inflammatory environment is not necessarily related to local genital tract environment and is not associated with HIV shedding [38].

Adaptive immunity is a pathogen-specific response after T-cell presentation and stimulation by antigen-presenting cells (APCs) or by B-cells antibodies secretion. This immune response is driven by APCs which, in the female genital tract, include macrophages, dendritic cells, Langerhans cells, and epithelial cells of the cervix and endometrium. The effector components are CD4^+^ T-cells as well as the cytokines they secrete, CD8^+^ T-cells (cytotoxic effector cells) and immunoglobulins. CD4^+^ T-cells are usually subdivided into Th1, Th2, and Treg and Th17 cells, whose development, in general, is directly or indirectly mediated by cytokines during antigen stimulation and which is influenced by hormones [39–43].

Th1 cell-mediated immunity involves the destruction of intracellular pathogens by macrophages and cytotoxic effector CD8^+^ T-cells activation, which destroy pathogens by recognizing antimicrobial peptide-expressing cells associated with MHCI, hence inducing apoptosis through perforin and granzymes [44]. CD4^+^ T-cells secrete high levels of IFN-*γ*, a cytokine that stimulates activation of CD8^+^ T-cells, leading to destruction of virus-infected cells [27, 45]. In female reproductive tract, CD8^+^ T-cells predominate over CD4^+^ T-cells [46, 47]. During the secretory phase of the cycle, when ovulation and implantation can occur, cellular immunity in the uterus achieves more immune modulation and impact in HIV susceptibility [15, 48–52].

Humoral immunity is characterized by the production of antigen-binding antibodies, thus allowing phagocytosis and inducing antibody-dependent cell-mediated cytotoxicity (ADCC) or eliminating the antigens through the complement system. The immunoglobulins found in the mucosa of the genital tract are, primarily, IgG and IgA; traditionally, IgA is the major immunoglobulin isotype found in secretions [53]. However, a predominance of IgG in relation to IgA has been observed in FRT secretions [54, 55]. The amount of immunoglobulins present in cervicovaginal secretions is strongly regulated by hormones, so it varies during the menstrual cycle, with a marked decrease in levels during ovulation [56]. According to Shrier et al. [57], IgA and IgG levels decrease during the follicular phase, reach a minimum amount during ovulation, and increase during the luteal phase. This particular feature may function as a mechanism which facilitates the survival of sperm in the genital mucosa and ensures efficiency of fertilization. However, during this period, alterations in immune functions could impact increased susceptibility or altered response to some infections [57–63].

Although B-cells that synthesize and secrete immunoglobulin are abundant in the endocervix, they are scanty in the vagina, hence suggesting the existence of cellular microenvironments distributed in the genital mucosa. It is possible that humoral immunity in the vagina canal is promoted by local production of immunoglobulins and their transport from the bloodstream to uterine mucosa [54].

As in the intestinal mucosa, IgA is found in the polymeric form (pIgA) in the genital mucosa, and it is transported to the lumen via polymeric immunoglobulin receptor (pIgR) in the secreted form of IgA (S-IgA). PIgR expression in the epithelium of the female genital tract is upregulated by cytokines from activated T-cells, such as IFN-*γ*, and also by hormonal changes [54]. Variations in pIgR expression by epithelial cells, caused by female sex hormones, may explain the differences in immunoglobulin levels in cervicovaginal secretions during the menstrual cycle [64]. However, IgG is a monomeric immunoglobulin, and it is probably not transported via pIgR. Nevertheless, IgG is also found in mucous secretions and has a key role in host immune response [65]. The role of IgG in mucosal secretions is controversial, since this isotype can act both as a protective mechanism and as a cytotoxic mediator due to its ability to activate complement proteins and induce antibody-mediated cytotoxicity via polymorphonuclear leukocyte Fc receptor, which would damage mucosal epithelium [54].

Recent studies indicate that antibodies in female reproductive tract from healthy HIV(+) and HIV(−) inhibit HIV infection and may play a role in the inhibition of HIV transmission [66]. Several reports have pointed out that IgA in genital mucosal can have an important role in inhibiting HIV transmission [67–70]. Although vaginal secretions of HIV-infected women exhibit IgG directed to a broad range of Env antigens and IgA reactivity to gp41, secretions of women that remain uninfected despite ongoing exposure to HIV-1 contain IgA mostly directed at HIV-1 gp120/gp140. These mucosal HIV-1-specific IgA antibodies could contribute to the protection of these women or be a marker of another protective function [71]. Besides these properties, antibodies in genital mucosa have several functions in the anti-HIV immune response, such as secretory IgA aggregation, Fc-mediated inhibition, neutralization of CD4 T cell infection, lysis of infected cells by NK cells, phagocytosis after antigen presentation, and inhibition following cytokine and chemokine production [72]. Most HIV-1 vaccination strategies aim to induce human HIV-specific antibodies able to inhibit the infection of target cells at the onset of viral transmission; however, inducing such bNAbs by vaccination is likely to be very difficult [73–75]. As HIV transmission at mucosal sites involves specific HIV targets, vaccination should induce an immune response that protects all the different potential mucosal target cells, and strategies to develop local immune responses should therefore be encouraged, leading to a strong and long-lasting response [72].

## 3. HIV/AIDS and Cells of the Female Genital Tract

Sexual transmission is the main route of transmission of HIV [76], with an increased risk of infection in women in comparison to men [76, 77]. Several characteristics of FRT make it susceptible to colonization, to the establishment of infection, and to the systemic spread of the virus, which causes local changes that may eventually facilitate infection by other microorganisms. During the infection establishment, the virus comes into contact with characteristic cells of the female genital tract—such as epithelial cells—and also with resident cells of the innate immune system, adaptive immune system, and mucosal-associated lymphoid tissue (MALT).

Despite being a subject of intensive studies, it is not fully elucidated what exactly occurs after HIV contact with the mucosa of female genital tract. Nevertheless, it is well established, especially in experimental models using Simian Immunodeficiency Virus (SIV) in* Rhesus* monkeys, that the infection starts with a small local virus population in the genital tract, which evolves into a marked systemic infection in a few weeks [78, 79]. Even though, conceptually, the local immune system is the first line of defense against infections, the induction of inflammatory process acts as a viral spread factor, so the activation of cells of the local immune system is a crucial factor for HIV establishment.

It is important to note that, at the cellular level, HIV infection occurs through interaction with receptors and coreceptors present in many cell types. The primary receptor for HIV entrance into a host cell is CD4, and the chemokine receptors CXCR4 and CCR5 are the major coreceptors [80–82]. Two main strains of HIV are described based on coreceptor usage, R5 and X4. Furthermore, several other receptors have been described as essential for HIV internalization and/or transfer between cells [83], such as mannose receptors, DC-SIGN, gal ceramide, heparan sulfate proteoglycans (HSPG), *α*4*β*7 integrin, and gp340 [84–87].

Local epithelial cells play an important role in the development of infection. Changes in epithelial thickness due to hormonal influence during the menstrual cycle, administration of local or systemic, hormonal or nonhormonal contraceptive methods [88–90], and menopause [91, 92] appear to influence the permissiveness to HIV infection, although further studies in humans are needed to determine this influence* in vivo*.

In general, three main mechanisms are associated with HIV migration through the epithelial cells of the female genital tract: through gaps or microlesions in the epithelial barrier; through transcytosis, which may be mediated by neonatal Fc receptor (FcRn); or through paracellular movement between epithelial cells [50]. Moreover, despite not being primary targets of HIV invasion, epithelial cells of the female genital tract are found to be permissive to HIV infection* in vitro*, although this phenomenon is still controversial* in vivo* [93–99].

*In vitro* studies also indicate that epithelial cells of the genital tract respond differently to R5 and X4 strains of HIV through cytokine production [98, 100–104] and also by driving cytokine production by other immune system cells, especially macrophages, which may interfere with the ability of these cells to recruit CD4^+^ T-cells [98].

Even though epithelial cells do not appear to be able to generate new virions under normal conditions, they can function as a virus transmission mechanism to permissive cells through HIV contact and transfer, thus mediating the systemic spread [105]. Recently infected cells have been detected in the cervical submucosa 3-4 days after primary infection and, in this initial period, the mucus in this region can act as a protective, although relatively inefficient, factor [106]. Furthermore, the presence of HIV directly affects the integrity of the epithelial barrier in the female genital tract mucosa, allowing for translocation of pathogens [107].

Dendritic cells (DCs) are believed to be the primary target of HIV infection, spreading the infection to the associated lymph nodes and acting as a disseminator [108–111]. Moreover, these cells can invaginate into the epithelium towards the lumen, capturing HIV and allowing its transmission to the submucosal cells, that is, phagocytes and CD4^+^ T-cells [109]. However, these cells also act directing anti-HIV adaptive immune response and priming CD4^+^ and CD8^+^ T-cells. Viral receptors present on DCs, such as TLR-8 and DC-SIGN, have been associated with the spread of HIV to CD4^+^ T-cells [112]. On the other hand, α-defensins 1-3 production by DCs is associated with a slower progression of HIV infection [113]. Moreover, several studies have pointed to an important interaction of HIV with a specific type of dendritic cells called Langerhans cells (LC), which are characterized by the expression of Langerin and by being found mainly in mucosae; they can get infected with HIV and act as a viral inhibitor [114], although they seem to act more as a mechanism of capture and transport of viruses to CD4^+^ T-cells [21, 115–117]. HIV infection leads to a reduction in LC density in the female genital mucosa [118], and the systemic activity of HIV infection also affects the population of LCs in the vagina, which decreases in the presence of detectable HIV-RNA but is not decreased in the absence of detectable plasma levels of HIV-RNA [119].

Among other cellular components of innate immunity in the female genital tract mucosa, macrophages participate actively in HIV infection; they are effector cells and one of the main targets of this virus. HIV infection leads to changes in macrophages both systemically and in the female genital tract mucosa. In general, HIV-infected monocytes and macrophages have several impaired or modulated functions which promote viral persistence or delay the development of the adaptive immune system, for instance, impaired antigen-presenting ability [120], impaired T-cell-activation ability [121, 122], impaired ability to engulf and destroy intracellular microorganisms [123–126], decreasing expression of surface molecules, such as CD36 [127], decreasing expression of Fc receptors [128, 129], impaired TLR signaling response [130], and increased susceptibility to phagocytosis [131].

Several studies have reported contradictory outcomes on the role of macrophages as cells promoting virus spread through vaginal mucosa transmission, especially when compared to* in vitro* studies with HIV-infected cells and SIV infection models. If, on the one hand, the authors indicate that the initial establishment of HIV infection is sustained by macrophages [132–134], infection of* Rhesus *monkeys with SIV failed to show this relationship, so it is required to determine the actual role of these cells as protagonists in the establishment of the infection [135]. However, despite such discrepancies, these cells have been continuously implicated as carriers of infection to CD4^+^ T-cells, to local cells, and to associated lymphoid tissues [112, 136]. A feature that may facilitate this process is the increased expression of CD4, CXCR4, and CCR5 receptors associated with HIV infection in vaginal macrophages rather than with other mucosae [137, 138].

On the other hand, studies have shown that these cells produce cytokines and chemokines that act as promoters of intense inflammatory infiltration in the mucosal lining of the female genital tract [139]. This inflammation plays a paradoxical role; it is, at the same time, an effector mechanism of antiviral immunity and one of the main promoters of the establishment of HIV infection, allowing the virus to multiplicate quickly and to infect new susceptible cells [139]. Indeed, enhanced HIV-1 replication in* ex vivo* ectocervical tissues from postmenopausal women correlates with increased inflammatory responses [140]. In this context, there is a marked induction of CCL2, CCL3, CCL4, CCL5, CXCL8, and CXCL10 after infection with SIV, as well as a systemic increase of CCL2, CXCL8, and CXCL10 in humans, concurrently with viremia peak after infection with HIV [141]. Once HIV is primarily concentrated in mucosae, they probably play a major role in the production of these mediators [78, 142]. These chemokines have a direct effect on the recruitment of new macrophages and CD4^+^ T-cells—targets of viral spread [79, 142–144]. Even though the local production of chemokines attracts more HIV target cells, chemokines may act as competitors for binding CCR5 and CXCR4 receptors [145, 146].

HIV interference in the macrophages functions, particularly in those related to viral persistence, is mainly mediated by Nef, which is a protein capable of modulating the surface receptors expression, interfering with intracellular signaling pathways, increasing the production of cytokines and chemokines, and changing phagocytic and autophagic capacity [147–152].

Although the relationship of HIV with the number of neutrophils and their functional impact is not well known and investigated, studies have pointed out that neutrophils are increased in the female genital tract in chronic infection due to the local increase of IL-1*β*, TNF-*α*, IL-8, IL-6, and IL-10 [153]. It still needs to be clarified whether this increase is, to some extent, a mechanism of cell control or recruitment for the perpetuation of infection. In a more general context, HIV infection has an impact on the formation of neutrophils in the bone marrow [154] and it reduces the expression of CD13 and CD16 [155, 156]. These cells have recently been implicated in the suppression of CD4^+^ T-cells via PD-L1/PD-1 [157] and in antibody-dependent cell death [158]. Moreover, hypochlorite/hypochlorous acid (HOCl) produced via myeloperoxidase (MPO) secretion to the outside of the cells has emerged as a mechanism of HIV destruction [159, 160]. On the other hand, HIV-1-infected peripheral blood mononuclear cells enhance neutrophil survival and HLA-DR expression via increased production of GM-CSF [161]. Recently, Neutrophil extracellular traps (NETs) have been described as a host defense response to human immunodeficiency virus-1 preventing virus spreading [162]. The recruitment of neutrophils and mononuclear phagocytes to an infectious site brings into play HNPs (human neutrophil peptide) and further increases in LL-37. Despite significant HIV inhibitory activity, cervicovaginal levels of *α*-defensins 1-3 (HNPs 1-3) and LL-37 were associated with increased HIV acquisition, perhaps due to their association with bacterial sexually transmitted infections [163]. On the other hand, human neutrophil *α*-defensin 4 inhibits HIV-1 infection* in vitro* [106].

Despite the clear involvement of NK cells in the immune response against the virus, classical studies have demonstrated that HIV-positive individuals have a poor response of these cells [164–166]. NK cells fail to degranulate and to produce IFN-*γ*, leading to poor response against bacteria [167], to human papillomavirus (HPV) [168, 169], and to an impaired antibody-dependent cell-mediated cytotoxicity associated with HIV infection progression [170]. Studies suggest that, at least in part, these defects can be related to functional depletion of NK cells, with possible involvement of PD-1 and TIM-3 [171, 172]. As previously discussed, female genital tract NK cells express different phenotypic markers from NK cells in the bloodstream, such as CD9, CD69 and CD94 [29]. It seems that uterine NK cells can inhibit the infection of target cells by HIV X4 but not R5 strains via the secretion of CXCL12 [29]. Functional modulation of NK cells has been correlated with resistance to HIV infection in highly exposed HIV-seronegative (HESN) [173]. Killer immunoglobulin-like receptors (KIRs) regulate natural killer (NK) cells in a human leukocyte antigen (HLA)—in a dependent manner, and KIR3DL1/S1 and KIR2DL2/DL3 loci have been linked to resistance to HIV infection [174, 175]. Also, KIR/HLA interactions may influence resistence versus susceptibility to virus transmission [175–179].

Among the cells in the mucosa of the genital tract, CD4^+^ T-cells seem to be the most important target for the establishment of a successful HIV infection [180]. Several studies have demonstrated that the infection starts with a small number of CD4^+^ T-cells infected with HIV that multiply before systemic viremia is established [181, 182]. As aforementioned, this key role is closely related to the presence of receptors and coreceptors that mediate the infection of these and other cells of the immune system. In fact, studies in humans have demonstrated that HIV infects, extremely effectively, CD4^+^ T-cells in several areas of the female genital tract, especially vagina, ectocervix, and endocervix [117].

Classic studies had already indicated that, besides the reduced CD4^+^ T cell count, several dysfunctions were observed in CD4^+^ T-cells from HIV-positive individuals, including an impaired response to polyclonal stimuli or recall antigens, reduced production of IFN-*γ* and IL-2, deficient TCR signaling pathways, and a proliferative capacity decrease [183–187].

Interestingly, studies have shown that different subtypes of CD4^+^ T-cells have different levels of susceptibility to HIV infection [117]. For instance, the T-cell subset expressing the integrin alpha4beta7—a mucosal homing receptor—is highly susceptible to HIV-1 infection [188]. Furthermore, most HIV infections through sexual intercourse apparently involve viruses that use CCR5, that is, R5 tropic strains, which implies that they preferentially infect cells that express this receptor [189–191]. Nevertheless, there is a significant population of CD4 T-cells expressing CXCR4 in the mucosa of the female genital tract, so its importance in the acquisition and development of infection needs to be clarified [192]. Interestingly, postmenopausal women show an increase in CD4^+^ CCR5^+^ T-cells in the gastrointestinal tract, which may lead to an increased susceptibility to HIV infection, even though the exact mechanism for this increase also needs to be elucidated [193].

Sexual transmission of human immunodeficiency virus type 1 (HIV-1) most often results from a productive infection by a single transmitted/founder (T/F) virus that rises to a productive systemic infection after approximately two weeks [194–198]. Recent reports have pointed out that T/F viruses utilize the CCR5 coreceptor for entry and replicate efficiently in cultures of activated primary T lymphocytes and replicate in monocyte-derived macrophages (MDM) [199]. Also, it seems that transmitted/founder and chronic HIV-1 are distinguished by differential utilization of CCR5—with T/F viruses presenting proteins with higher CCR affinity [200–202], favoring preferential sexual transmission of R5 strain.

Recent findings have supported the impression that, although HIV is virtually able to infect any CD4^+^ T cell, there is a preference for the infection of activated lymphocytes [181]. T-cell profiles with a more inflammatory nature, such as Th17, are preferably infected and allow for greater viral replication, resulting in depletion of these cells in HIV-infected individuals compared with healthy individuals and showing a proportional decrease in viral load [203–206]. Moreover, it was demonstrated that a subset of activated cervical CD4^+^ T-cells that expresses *α*4*β*7, CCR5, IL-17A, and IFN-*γ* preferentially binds HIV-1 gp120* in vitro*, and these cells are almost entirely depleted from the cervix* in vivo* during HIV infection. The presence of multiple susceptibility markers in a subset of CD4^+^ T-cells in the genital tract suggests that these cells may play a key role as HIV targets during sexual transmission [192].

Along with Th17 cells, IL-22-producing T-cells and Th22 cells were dramatically depleted during chronic HIV infection, concomitant with epithelial integrity impairment and increased microbial translocation [207]. Furthermore, circulating Th22 cells expressed a higher level of the HIV coreceptor/binding molecules CCR5 and *α*4*β*7 than CD4^+^ T-cell subsets in HIV-uninfected participants, but this was not the case after HIV infection [207]. The role of the differential infection of these T-cell profiles in HIV susceptibility and in other associated infections needs to be better clarified.

A recent review brought an interesting thought that could help explain the low infection-to-exposure ratio and the selection of the founder strain after sexual exposure to HIV [208]. The founder strain is a single virus variant that usually initiates the infection with HIV, despite the presence of thousands of genetically diverse viral strains in the transmitting partner [190, 209, 210]. Comparing this situation with the transmission dynamics of infectious diseases, where differences in the ability of individuals to spread the infection may set whether an outbreak will turn into an epidemic [211], and based on evidence from molecular biology and virology, the authors suggest that heterogeneity among CD4^+^ T-cells could yield wide variation in the capability of individual cells of becoming infected and of transmitting HIV to other cells [208]. Using an epidemiological framework, they suggest that such heterogeneity among CD4^+^T may play a critical role in the establishment and spread of HIV in the genital mucosa after sexual exposure [208]. Although there are no* in vivo* evidence that this phenomenon occurs (despite differences in subsets CD4^+^ T-cells in susceptibility to HIV infection, as discussed above), it may explain why some women might be more susceptible to HIV sexual transmission than others.

After the establishment of HIV infection in the genital mucosa, the presence of cytotoxic T-cells is detected not only in this region but also in mucous membranes in general. Nevertheless, as previously suggested, the response of these cells is “too little and too late” to contain viral replication and dissemination [212]. A previous report showed that proinflammatory cytokines in the female genital tract may promote HIV replication and shedding. These cytokines were associated with increased levels of HIV-specific CD8 effector cells in the genital mucosa but these were not able to control genital HIV shedding [213].

As the infection progresses, CD8^+^ T-cells can be detected in the genital tract mucosa of HIV-positive women [213–215]. Although, in general, CD8^+^ T-cells from healthy individuals are polyfunctional, the ability of cervical CD8^+^ T-cells to exhibit polyfunctional responses (ability to produce 2 or more cytokines) following HIV stimulation is inversely associated with plasma viral load and positively associated with blood CD4 counts, suggesting that clinical status had an impact on the functionality of HIV-specific T-cells in the mucosa and that cervical T-cells are largely monofunctional. Furthermore, polyfunctional T-cells in the cervix of women with high blood CD4 count and low plasma viral load do not protect from HIV genital shedding [216].

## 4. Immunity of Female Genital Mucosa, HIV/AIDS and STDs

### 4.1. Bacterial Vaginosis and HIV/AIDS

Bacterial vaginosis is a condition caused by an alteration in the genital tract flora in which lactobacilli are predominantly replaced by anaerobic bacteria, including* Gardnerella vaginalis*,* Mycoplasma hominis*, and* Atopobium vaginae* as well as species of* Prevotella, Mobiluncus*, and* Peptostreptococcus* [217]. Considered the most common cause of vaginitis, bacterial vaginosis is associated with urinary tract infections, pelvic inflammatory disease, preterm labor, and also an increased risk of HIV infection [218, 219]. The incidence of bacterial vaginosis ranges from 8% to 23% of women of reproductive age, whereas the incidence rate is as high as approximately 50% of HIV-infected women [220, 221].

The lactobacilli present in a healthy vaginal microbiome, especially* Lactobacillus acidophilus*, predominantly produce hydrogen peroxide (H_2_O_2_), which is responsible for maintaining a low vaginal pH and for inhibiting microbial growth, including the causative agents of bacterial vaginosis [222]. H_2_O_2_ also has a virucidal effect on HIV-1 [223]; it inhibits viral adhesion and replication, as well as CD4^+^ T cell activation, therefore reducing the number of HIV target cells in the vagina [224].

Like other sexually transmitted pathogens, bacterial vaginosis has been associated with increased acquisition and transmission of HIV in women [221, 225]. Studies have shown that an increased HIV shedding is found in vaginal secretions of women with bacterial vaginosis or with low levels of lactobacilli [226, 227] and also that these secretions/fluids are able to stimulate HIV expression* in vitro* [164].

The immunosuppression caused by HIV can increase the frequency or severity of bacterial vaginosis. The disease is more persistent among HIV-infected women with lower CD4 cell counts who are also more likely to develop more severe symptoms of bacterial vaginosis [220]. Analyses of vaginal secretions of HIV-seropositive women demonstrated an inverse correlation between viral load and lactobacillus species and a positive correlation between bacterial vaginosis and* Mycoplasma hominis* [228].

Previous studies suggested that anaerobic bacterial growth and increased pH observed in the establishment of bacterial vaginosis profile inhibit the concomitant growth of* Candida* in these patients [229]. Bacterial vaginosis also influences the development of cervical intraepithelial neoplasia, possibly due to the production of nitrosamines that can cause DNA damage and to the change in the profile of cytokines that decrease the ability of the immune system to eliminate HPV infection [230]. Furthermore, HIV infection itself alters the vaginal immune response and the cytokine profile, further increasing the etiologic mechanisms between this association of bacterial vaginosis and precancerous lesions [231].

### 4.2. Candidiasis and HIV/AIDS

*Candida albicans*, the main causative agent of vaginal candidiasis, colonizes the genital tract of 75% of women of reproductive age, regardless of sexual activity [232], and about 5% to 8% of these women experience recurrent infection [233].

HIV infection has been considered one of the risk factors for the development of symptomatic* Candida* infection, together with pregnancy, uncontrolled diabetes mellitus, and use of corticosteroids, antibiotics, or oral contraceptive pills with high estrogen concentrations [234].

In the HIV/AIDS scenario, mucosal candidiasis is one of the oldest and most common opportunistic infections in HIV-positive women [235, 236]. Although it is not considered a syndrome-defining illness, vaginal candidiasis is classified by the centers for disease control and prevention for human immunodeficiency virus as a condition whose course or management may be altered by HIV infection [237–240].

Although a higher frequency of vulvovaginitis in women infected with HIV is not yet fully established, most studies show a higher prevalence of vaginal candidiasis with a higher recurrence risk in HIV-infected women than in uninfected women [236, 239, 241–243]. Other authors also claim that the increased rates of* Candida* colonization are associated with decreased CD4^+^ T cell count, especially below 200 cells/mm^3^ [241, 244, 245], indicative of systemic immune failure.

HIV infection is clearly involved in changes in the normal vaginal flora that favor the development of local infections and sexually transmitted diseases, which, in addition to increasing local replication of the virus, potentially play a role in the sexual transmission of HIV [246, 247]. This is because HIV infection reduces the number of CD4^+^ T-cells and leads to increased chemokine levels in the vaginal mucosa [242, 248], whereas vaginal infections increase susceptibility to virus infection due to a lower production of hydrogen peroxide by lactobacilli and to the disruption of the normal epithelial barrier [249]. On the other hand, as previously discussed, inflammatory stimuli and consequent immune activation could act as a direct mechanism that attracts target cells and favors HIV replication [139, 140].

The symptoms of vaginal candidiasis often occur at an earlier stage in the course of HIV infection, and they are associated with the reduction of the normal vaginal flora and pH, favoring germ tube formation in yeast cells and increased inflammation and virulence [250], even though the severity of the infection is not increased in HIV-positive women. Furthermore, after vaginal infection treatment, the recurrence time is higher in HIV-positive patients, and the time interval correlates closely with the severity of immunosuppression [251].

### 4.3. Trichomonas Vaginalis and HIV/AIDS

Trichomoniasis, which is caused by the flagellated protozoan* Trichomonas vaginalis*, is one of the most common sexually transmitted diseases (STDs) in HIV-infected women and uninfected women. Epidemiological studies show prevalence rates ranging from 6% to 27% in the group of HIV-positive women, with values reaching 36% in reinfection episodes by the protozoan [220, 252, 253].

This STD is associated with pregnancy complications such as premature rupture of membranes and premature birth, infertility, and cervical cancer [254, 255]. The fact that HIV infection risk is 2–9 times higher in the presence of a sexually transmitted disease [256] has highlighted trichomoniasis as an important disease in the scenario of HIV/AIDS infection and transmission [257], and effective treatment of this disease is an important strategy for HIV prevention.

Trichomoniasis is considered a risk factor for HIV transmission and infection because it stimulates an intense inflammatory response of the vaginal epithelium and ectocervix with evidence of punctate hemorrhages, causing disruptions of the urogenital epithelium that act as virus gateways [258].

*Trichomonas* infection in HIV-positive women is associated with an increase in genital shedding of HIV [259] and, similarly to what happens with vaginal candidiasis in HIV-infected women, the prevalence of trichomoniasis increases with the decline in immune function measured by CD4^+^ T cell count and nonadherence to HAART [260].

### 4.4. Genital Herpes and HIV/AIDS

Genital herpes, caused by* Herpes simplex* type-2 (HSV-2), is a chronic sexually transmitted disease of high global burden that causes genital ulcers [261]. The incidence rates of genital herpes in HIV-positive women have been reported to vary between 11% and 50%; however, more than 80% of these patients are asymptomatic [262–264].

Considered one of the greatest risk factors for HIV acquisition in developing countries, HSV-2 seroprevalence rates reach 90–95% in HIV-infected women [265], and the frequency of reactivation of herpes simplex type-2 is also 2–4 times higher in this population than in uninfected women [266, 267].

As in diseases such as syphilis and chancroid, HSV-2 is particularly important in the acquisition and transmission of HIV since it allows for the disruption of mucosa causing inflammation and the development of ulcers, which facilitates HIV entry [268].

Coinfection between HIV and HSV-2 and the bidirectional synergistic relationship between them increase the spread of viruses causing AIDS and genital herpes. HIV infection increases genital shedding and the frequency of herpes reactivation, which, in turn, increases the concentrations of HIV in the plasma and in genital secretions and the risk of HIV transmission and disease progression [269, 270]. On the other hand, coinfection of CD4 T-cells by HIV and HSV results in a rapid unidirectional replication of HIV, whereas the acquisition, reactivation, and shedding of HSV-2 are facilitated by disturbances in the vaginal flora resulting in bacterial vaginosis [271, 272].

The frequency and severity of HSV-2 recurrences are enhanced by the immunodeficiency caused by HIV infection. Even though treatment with high potency antiretroviral therapy reduces the frequency and severity of genital herpes, HIV-seropositive women have a higher number of genital ulcers and increased viral shedding than uninfected women [273, 274]. In general, genital herpes ulcers are more generalized, painful, persistent, and recurrent in HIV-positive patients [275].

### 4.5. Pelvic Inflammatory Disease (PID) and HIV/AIDS

Pelvic inflammatory disease (PID) comprises a spectrum of inflammatory disorders of the female upper genital tract that are caused by microorganisms arising from the uterine cervix [276]. The signs and symptoms are nonspecific and varied, and 70–80% of the cases are asymptomatic [277]. Classically associated infectious agents include* Chlamydia trachomatis* and* Neisseria gonorrhoeae*.

*Chlamydia* is the most common sexually transmitted disease (STD) in the world, more common than syphilis and gonorrhea [278]. It is an obligate intracellular bacterium that causes polymorphonuclear infiltration in the epithelium at the onset of infection, followed by subepithelial mononuclear infiltration with tissue injury and scarring, which leads to the following frequent sequelae, infertility, ectopic pregnancy [279], and chronic pelvic pain.

This bacterium has several factors involved in cytokine production through the stimulation of TLRs [280], HSP60 [281], and chlamydial CpG-DNA [282]. Along with TNF-*α*, IL-1 (a cytokine produced by infected epithelial cells) induces the production of IL-8 by endothelial cells, epithelial cells, and monocytes; the latter are the major neutrophil chemotactic factor [279]. Furthermore, IL-1 binding to its respective receptor initiates a mitogen-activated protein (MAP) kinases pathway, such as the p38 pathway, which is also activated by binding to TLRs and to the TNF receptor, and which will promote the transcription of adhesion molecules [283].

The recruitment of leukocytes in genital inflammatory diseases such as infections caused by* C. trachomatis* and* N. gonorrhoeae* enhances the acquisition and transmission of HIV [284], with an increase in the concentration of HIV-1 in the genital secretion. Similarly, the significant epithelial lesion in cases of* Chlamydia* infection is definitely a contributing factor in coinfection [285].

Moreover, proinflammatory cytokines generally stimulate replication of HIV virus in the genital tract, as they activate nuclear factor *κ*B as well as recruit and activate leukocytes, which are the targets of the virus [153]. Cytokines such as TNF-*α* can permeabilize the epithelial intercellular junctions, facilitating HIV infection [107]. The exact definition of the role of each inflammatory mediator is still divergent in the literature, probably due to the difference in the cohort studied and to the various factors involved.

Baeten et al. recently revealed a correlation between high levels of HIV-1 RNA in the genital secretion and an increased risk of transmission, regardless of the serum levels of HIV. Genital shedding of HIV1 can happen even in women with undetectable serum levels of HIV-1 [286], an occurrence which may be explained by the infections of the lower genital tract [38]. It is known that HAART (highly active antiretroviral therapy) reduces HIV-1 concentration in vaginal secretions [287] and in the plasma, as well as the risk of transmission [288, 289], but it does not eliminate it. An approach that integrates both treatments, HAART and a specific approach for each STD, is certainly more effective in controlling the disease. Nonetheless, because of insufficient monitoring or absence of symptoms in patients with AIDS, the major challenge is allowing for the early diagnosis of these STDs.

Diseases with genital discharge, such as gonorrhea, are particularly likely to increase HIV release in semen and cervicovaginal secretions [290]. Previous reports showed that gonorrhea is the sexually transmitted disease most associated with HIV, with an increment in the risk of HIV infection [248, 291–293].

*N. gonorrhoeae* activates TLR2 on CD4 T-cells, thus facilitating the infection, even at an earlier phase of the viral cycle, immediately after infection [292]. Furthermore, the bacterium increases the ability of the virus to infect dendritic cells, which, in turn, can introduce the virus to other susceptible cells [293].

### 4.6. Human Papillomavirus (HPV) and HIV/AIDS

There are about 40 genotypes of HPV capable of infecting the genital tract, and they are divided into two groups based on their oncogenic potential: low risk (causing genital warts/warts) and high risk [294], causing cervical cancer. Cervical cancer is the second most common cancer in women worldwide [295] and, in almost all cases, it is associated with persistent infection by high risk HPV types, especially 16 and 18 [296]. In turn, infection by HPV, a DNA virus, is the most common sexually transmitted disease in the world [297], and it causes anogenital lesions that are more prevalent in women. High risk HPVs are also associated with vulvar, vaginal, penile, anal, and oropharyngeal cancers [298]. Sexually active men seem to be a reservoir of high risk HPV subtypes [299].

The virus infects basal epithelial cells through microabrasions on the epithelial surface. The oncogenesis depends on the integration of the viral episome into the DNA of the epithelial cell [300] and on the activity of the viral oncoproteins E6 and E7 [301]. Both CD4^+^ and CD8^+^ T-cells are capable of recognizing these proteins. E6 is responsible for the degradation of proapoptotic proteins such as p53 [302], Mantovani and Banks [303]; whereas E7 promotes cell entry into the S phase of the cell cycle [304], and it integrates with the tumor suppressor protein pRb [305]. There are few copies of the viral genome in the basal layer [304]; the production of infective particles occurs only in the more differentiated layers of the epithelium [306].

Seroconversion occurs in 60% of immunocompetent women, but antibody production during infection is not able to prevent reinfections, probably due to the low titers [307].

The cellular immune response against viral proteins in immunocompetent individuals is responsible for suppressing the infection in 90% of patients in two years [308], with regression of premalignant and low grade squamous intraepithelial lesions, even without therapeutic interventions. In individuals with HIV/AIDS, this clearance is impaired [309].

The development of HPV-16 associated lesions, the most common high risk HPV subtype and that has early progression [302], is connected with the ineffective immune response against HPV-16 E6 and E7 [310]. The immune status also appears to influence the prevalence of HPV-18, which is between 15% and 18% in immunocompetent women and approximately 80% in immunocompromised women [300].

In persistent HPV infection there is no significant local inflammatory reaction, and therefore there is no significant increase in the risk of HIV acquisition/transmission in the genital tract [311]. Nonetheless, in nonpersistent infections, there is an increased number of T-cells in the infected cervix [312]. As with the majority of intracellular viruses, these cells are necessary to overcome the infection, and, because they are HIV targets, there is a transient increase in the risk of HIV infection during the regression process of HPV lesions [313]. Another potential mechanism is that the E7 protein of HPV-16 decreases the expression of E-cadherin, an epithelial adhesion molecule, hence increasing the permeability of the epithelium to HIV [314]. The meta-analysis performed by Houlihan et al. indicated a twofold increase in the risk of HIV infection when there was prior infection by any HPV genotype [294].

Furthermore, HIV coinfection increases the detection, persistence, and severity of HPV lesions [315]. This is caused by immunosuppression, which generates persistent HPV infection, infection by subtypes that are more susceptible to cause carcinomas, simultaneous infection by several subtypes, reactivations, and infection by HPV in several sites in the anogenital region [302]. In HIV-infected women, cervical cancer behaves more aggressively, with more recurrences and a worse prognosis, as well as being somewhat responsive to traditional therapies [316].

## 5. Conclusion

The female genital tract mucosa has an inflammatory environment somewhat independent from the systemic environment concerning HIV shedding. Several studies showed genital HIV shedding in women with undetectable plasma viral load. This occurrence is due to local immune particularities and mainly because of coinfections by other sexually transmitted diseases. There is a bidirectional synergistic relationship in which HIV infection favors the development of local infections and these diseases increase local replication of the virus.

Certainly, an adequate approach to control this process would be the combination of HAART and the specific treatment of each sexually transmitted disease. The great challenge is to diagnose these conditions, as patients with AIDS can be assintomatic. Therefore, rigorous monitoring is required.
